# Pan-Britain, mixed-methods study of multidisciplinary teams teaching parents to manage children's long-term kidney conditions at home: Study protocol

**DOI:** 10.1186/1472-6963-12-33

**Published:** 2012-02-14

**Authors:** Veronica M Swallow, Davina Allen, Julian Williams, Trish Smith, Jean Crosier, Heather Lambert, Leila Qizalbash, Lucy Wirz, Nicholas JA Webb

**Affiliations:** 1 School of Nursing, Midwifery and Social Work, University of Manchester, Oxford Road, Manchester, M13 9Pl, UK; 2 Royal Manchester Children's Hospital, Central Manchester University Hospitals NHS Foundation Trust, Oxford Road, Manchester, M13 9WL, UK; 3 The Great North Children's Hospital, Newcastle upon Tyne Hospitals NHS Foundation Trust, Queen Victoria Road, Newcastle upon Tyne, NE1 4LP, UK; 4 Cardiff School of Nursing and Midwifery Studies, Cardiff University, Newport Road, Cardiff, CF24 0AB, UK; 5 School of Education, University of Manchester, Oxford Road, Manchester, M13 9PL, UK

## Abstract

**Background:**

Care of children and young people (children) with long-term kidney conditions is usually managed by multidisciplinary teams. Published guidance recommends that whenever possible children with long-term conditions remain at home, meaning parents may be responsible for performing the majority of clinical care-giving. Multidisciplinary team members, therefore, spend considerable time promoting parents' learning about care-delivery and monitoring care-giving. However, this parent-educative aspect of clinicians' role is rarely articulated in the literature so little evidence exists to inform professionals' parent-teaching interventions.

**Methods/Design:**

This ongoing study addresses this issue using a combination of quantitative and qualitative methods involving the twelve children's kidney units in England, Scotland and Wales. Phase I involves a survey of multidisciplinary team members' parent-teaching interventions using:

i) A telephone-administered questionnaire to determine: the numbers of professionals from different disciplines in each team, the information/skills individual professionals relay to parents and the teaching strategies/interventions they use. Data will be managed using SPSS to produce descriptive statistics

ii) Digitally-recorded, qualitative group or individual interviews with multidisciplinary team members to explore their accounts of the parent-teaching component of their role. Interviews will be transcribed anonymously and analysed using Framework Technique. Sampling criteria will be derived from analysis to identify one/two unit(s) for subsequent in-depth study

Phase II involves six prospective, ethnographic case-studies of professional-parent interactions during parent-teaching encounters. Parents of six children with a long-term kidney condition will be purposively sampled according to their child's age, diagnosis, ethnicity and the clinical care-giving required; snowball sampling will identify the professionals involved in each case-study. Participants will provide signed consent; data gathering will involve a combination of: minimally-obtrusive observations in the clinical setting and families' homes; de-briefing interviews with participants to obtain views on selected interactions; focussed 'verbatim' field-notes, and case-note reviews. Data gathering will focus on communication between parents and professionals as parents learn care-giving skills and knowledge. Interviews will be digitally recorded and transcribed anonymously.

**Discussion:**

This study involves an iterative-inductive approach and will provide a unique, detailed insight into the social context in which professionals teach and parents learn; it will inform professionals' parent-educative roles, educational curricula, and health care policy

## Background

Care of children with long-term conditions in the children's kidney units in England, Scotland and Wales is managed by multidisciplinary teams (MDTs) comprising professionals such as clinical psychologists, dieticians, doctors, nurses, pharmacists, playworkers, social workers, and therapists. Within the constraints of treatment regimens it is believed to be in children's best interests for them to be cared for at home whenever possible [1-4]. Professionals in the units, therefore, spend considerable time teaching parents who are from a wide range of socioeconomic and educational backgrounds and who have different learning needs, to deliver home-based clinical care to their children. Some MDT members also visit the child's home/school to initiate training and provide parents with ongoing support and disease monitoring. Currently little guidance exits to inform professionals of the types of teaching and support parents prefer and the most effective ways to teach parents.

### Parents delivering home-based clinical care

Although some parents of children with long-term conditions readily accept the care-giving role and adapt to it by developing competent management styles involving mastery and routinisation of treatment that minimise the intrusiveness of conditions, others experience difficulties, and the condition remains an unwelcome focus of family life [4-6]. There is evidence that some parents find the relentless requirements of home-based care-giving difficult to maintain, either because they do not want to take on all aspects of care-giving or they lack the comprehension to understand health care instructions. In addition there is emerging evidence that, from the outset of the long-term condition trajectory some parents are reluctant to acknowledge any learning or comprehension difficulties in case professionals judge them to be incompetent parents and/or do not 'allow' them to take their child home. As negative clinical outcomes can occur if parents do not adequately manage home-based care, it is important to gain a more detailed understanding of the social context in which professionals teach and parents learn so that MDTs can use this information to inform the parent-teaching component of their role.

Parents increasingly... *perform the vast majority of care-giving, including tasks that are complex and demanding *[7:13] but if they are not competent care-givers, they may not adhere to treatment regimens or may fail to recognise subtle clinical changes [8,9] so negative outcomes such as undetected urinary tract infections, damaged kidneys, impaired kidney function, relapse of the condition, and transplant rejection may occur. All of these carry significant emotional, physical and financial costs for families [4,6], and have financial implications for the National Health Service (NHS) [7]. Moreover, the limited evidence of parents' clinical care-giving in long-term condition management that does exist draws on data collected from parents whose care-giving practices were already established, and who had consequently developed their own unique management styles [10-12]. However, little prospective evidence exists to tell us about early development of the parental care-giving role from parents' or professionals' perspectives.

### Integrated working between professionals and parents

Delivery of high quality care for children with kidney conditions [13] requires integrated working between healthcare professionals, close working relationships with primary care teams, liaison with other healthcare teams and outside agencies and the sharing of skills and knowledge between MDTs and parents. Moreover, parents need access to accurate and accessible information in order to make informed decisions in partnership with clinicians and an agreed care plan that promotes children's best possible quality of life [7]. When managing children's conditions MDTs spend considerable time educating parents about the condition and facilitating their home-based clinical care-giving [6,14]. Although few data exist relating to MDT management of children's renal conditions a recent retrospective case-note review of 44 American children with renal insufficiency demonstrated better clinical outcomes for those managed in an MDT clinic compared to those managed in a general nephrology clinic [15]. However, the strategies MDTs use to educate parents from early in the renal journey and the types of teaching that parents prefer have received little attention in the literature.

### Links with our previous research

Members of the current research team recently undertook qualitative studies that explored family learning in one children's kidney unit in England. Parents and professionals' described the way parents' learned to: collect and test urine; understand investigations; administer specialist diets, medications, gastrostomy or naso-gastric tube-feeds; manage peritoneal dialysis; monitor diet and fluids; recognise the importance of subtle clinical changes; record clinical observations; act on observations and results, and accurately communicate observations/actions to professionals [6,14,16,17]. During this shared practice parents adopted the identity of 'students' needing to learn new skills, while professionals such as nurses functioned as 'family learning brokers' who demonstrated five distinct yet overlapping teaching activities: assessing parents' learning needs, creating learning opportunities, implementing teaching strategies, acting as interpreters and ambassadors, and assessing learning progress. Over time many parents successfully and independently managed care-giving but some reported negative emotional and physiological responses to the relentless responsibility. Moreover, within couples fathers' and mothers' views were sometimes at variance; this finding corresponds with earlier reports that also highlight the dearth of research focussing on fathers' viewpoints in child-health care and the fact that fathers' views are often underrepresented in clinical research [18,19-23].

To generate knowledge about the contributions both fathers and mothers make to management of children's long-term conditions we later conducted a qualitative study of fathers' and mothers' individual and joint accounts of care-giving in one children's kidney unit. We found that fathers and mothers made a significant contribution to management and a key theme identified was 'developing skills' in: information processing, sharing/negotiating care giving, restraining children, adapting to treatment regimens and communicating. Although skill development was often a challenging and uncertain process, fathers and mothers often negotiated care-giving with each other to accommodate this while caring for other children, undertaking paid employment and providing mutual practical and emotional support. Developing skills in holding their child for procedures and treatments was a major concern, but it was fathers who assumed the 'protector' role and worried more about their child's long-term health and well-being, while mothers concerned themselves more with current clinical issues and maintaining relationships with professionals [24]. Fathers also reported a preference for receiving information 'first hand' from professionals rather than 'second hand' from the mother. Meanwhile, we conducted a narrative review of 29 studies involving fathers in health care delivery. The studies were carried out in Australia (2), Canada (6), China (1), Israel (1), Taiwan (1), UK (3), USA (14), UK and USA (1). The review demonstrates that fathers' involvement in children's health care can positively impact on fathers', mothers' and children's well-being and family functioning [25]. Both fathers' and mothers' accounts of clinical care-giving are, therefore, important targets for future research.

A limitation of previous studies, including our own, is that they were not able to focus on observations of actual encounters between MDT members and parents at times when parents were being trained to become care-givers. Therefore, detailed, prospective research is needed to investigate the ways professionals promote mothers' and fathers' learning from early in the parents' care-giving journey. This will help MDTs to promote safe and effective home-based management of children's conditions, thereby contributing to optimum clinical outcomes. This paper describes the design of a prospective study that seeks to address this important gap in our understanding of professional's parent-educative functions.

### Research aim and objectives

The aim of this study is to obtain a detailed understanding of the way multidisciplinary teams teach fathers and mothers to become home-based care-givers for children's long term kidney conditions.

The objectives are to:

• Develop a descriptive profile of multidisciplinary team members' parent-teaching interventions in the children's kidney units in England, Scotland and Wales

• Explore MDT members' detailed accounts of the range of care-giving skills and information they relay to parents, and the formal/informal teaching strategies and interventions they use.

• Obtain a focussed and detailed prospective understanding of professional-parent interactions during observation of planned and ad hoc teaching and learning encounters in one or two units.

## Methods/Design

Parent education is a complex, multidimensional process representing a variety of cultural, cognitive, social and emotional factors so a single methodological approach would not yield meaningful data [26,27]. To achieve breadth and depth of analysis the study uses a combination of quantitative and qualitative methods [28]. To ensure rigour we will regularly review our methods using recognised check-lists for mixed methods and qualitative research [29,30]

### Study setting

Twelve children's kidney units in England, Scotland and Wales

### Phase I: Pan-Britain mixed methods survey

#### (i) Administered questionnaire

**Figure 1:**
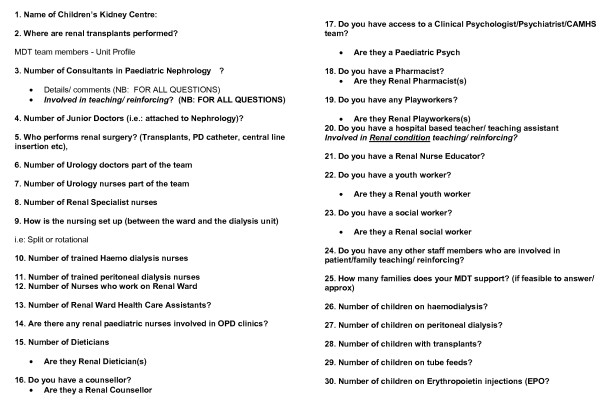
**Phase 1 questionnaire**.

A questionnaire (Figure 1) comprising a range of closed questions to survey: the numbers of professionals from different disciplines in each team, the information/skills individual professionals relay to parents, the teaching and support interventions they use, the existing patient categories by diagnosis and treatment support needed by parents (eg post-transplant care, or management of haemo dialysis, peritoneal dialysis, dietary restrictions, injections, naso-gastric tube feeding and complex medications) and the disciplines represented within the MDTs. Questions about the MDT include the number of: Consultants in Paediatric Nephrology; junior doctors (i.e: attached to Nephrology); urology doctors as part of the team; urology nurses as part of the team; Renal Specialist nurses; trained haemo dialysis nurses; trained peritoneal dialysis nurses; nurses or healthcare assistants who work on the Renal Ward or Renal Outpatients Clinic; Renal Dietician(s); Counsellor(s); Clinical Psychologist(s); Pharmacist(s); Play worker(s); Social worker(s); hospital-based teacher/teaching assistant(s); Renal nurse educator(s), Youth worker(s). Additional questions enquire whether individuals teach parents, reinforce information taught by colleagues or teach and reinforce.

The lead clinician/principle investigator (PI) in each unit (identified through the British Association for Paediatric Nephrology) or a delegated colleague completes one or two booked telephone interview(s) lasting 15-30 minutes at a convenient date/time. Data collection involves completion, by the researcher appointed, of the administered questionnaire. Telephone interviews combined with administered questionnaires are an effective means of surveying busy clinicians [31,32], and can result in lower 'missing-response' rates and less use of 'don't-know' options than postal questionnaires. Data are entered into SPSS to produce descriptive statistics.

#### (ii) Qualitative focus groups

##### 1. Participant selection and data collection

The PI or delegated colleague in each unit circulates study information to the MDT members in their respective units. Interested professionals contact the researcher by email or telephone for further information and/or to participate in a focus group or individual interview. The researcher organises venues and mutually convenient dates and times to visit each unit to conduct digitally-recorded focus-group interviews/individual interviews with consenting professionals. Participants can choose individual interviews if these are more convenient or they feel uncomfortable discussing their teaching in front of colleagues. Discussions, based on a topic guide derived from the literature and our previous studies and our clinical experience, explore in detail the range of care-giving skills/information individuals relay to parents, and the formal/informal teaching and support interventions they use. Focus groups and interviews are digitally recorded and transcribed verbatim.

##### 3. Data analysis

Data analysis and management involves Framework Technique and Excel [26,33]. Framework borrows its principles and approach to implementing these principles from different epistemological traditions within the social sciences; it is this eclecticism that has remained its strength throughout its development as an analytical process [26,34]. Framework's ontological position is closely related to subtle realism [35] which accepts that the social world does exist independently of individual subjective understanding, but that it is only accessible in qualitative research via participants' interpretations which may then be further interpreted by the researcher. Framework is systematic, thorough and grounded in the data but also flexible and enables easy retrieval of data to show others. This technique allows between and within case analysis and involves a process of familiarisation with data, identification of recurrent themes, indexing, charting, abstraction and interpretation. The iterative process of data management involves two researchers moving backwards and forwards between the stages of Framework. After closely reading all transcripts, a coding framework of themes and sub-themes is developed and then applied manually to all transcripts (indexing); data summaries and key direct quotations are labelled using the numerical reference to the appropriate theme/sub-theme and then 'lifted' to a Microsoft Excel spreadsheet for charting and referenced back to the page number on the original transcript for later retrieval when reporting. This charting process enables comparison within and between cases and rearrangement of the data case-by-case and by thematic content for subsequent descriptive analysis and abstraction. Working through the raw data with this level of intensity helps researchers to identify further lines of enquiry to pursue during descriptive analysis, the main purpose being to unpack the content and nature of each theme and to display the data in a way that makes distinctions that are meaningful and to provide content that is illuminating. At the first stage of abstraction descriptions are kept close to the original data so that the initial elements can be seen within the audit trail. In the more abstract categorizations different classifications can emerge. By retaining the connection between original data and the categorizations in this way, data will remain available for retrieval later in the analysis if needed [27]. During this process the substantive content and elements of each theme are identified. Several authors then work independently with data samples, searching for patterns within the data, mapping connections and seeking explanations for patterns before comparing and discussing these collectively until achieving a consensus. Constant comparison between transcripts helps open up meaning in the text until no new themes emerge, by which time data saturation is achieved. Sampling criteria derived from the analysis leads to identification of one/two unit(s) for in-depth study during phase II.

### Phase II: Focussed ethnographic study

In this phase we are gathering detailed prospective information on how MDT members promote parents' knowledge and skills development. This is achieved by carrying out five or six observational case-studies in the unit(s) selected using an ethnographic approach [35-37] involving detailed observation of behaviours and talk [38], Each case study lasts approximately six months. Data gathered provides information on how professional-parent interaction is enacted and identifies the communication processes that appear to optimise or impede professionals' contributions to parents' learning. The researcher will spend considerable time in the unit(s) to become familiar with the setting and in order to be sensitive to the clinical context surrounding ill children, parents and busy professionals. Studies of children about to embark on a new clinical intervention(s) and who are expected to require regular home-based care-giving such as: collecting/testing urine samples, understanding investigations, administering medications, dietary supplements, gastrostomy or naso-gastric tube feeds, setting up/running peritoneal dialysis, monitoring diet and fluids, recognising subtle clinical changes, recording clinical observations, acting on results and accurately communicating observations/actions to professionals, are being undertaken.

#### 1. Sample selection and recruitment

Sampling involves a maximum variation, purposive sampling approach based on the child's age (for example two children aged 0-5 yrs, two aged 6-11 years and two aged 12-18 years to allow for broad representation of stages of cognitive development as it is recognised that considerable differences exist between care needs of children and young people of different ages) [39,40], sex, ethnicity and type of care-giving. Children are identified by the local PI. Snowball sampling identifies the MDT members involved in each case. As each child and parents are recruited, access negotiations commence on an individual basis with MDT members expected to be involved in the child's care. In addition to parental consent, assent is required from children thought to possess the competence needed to understand the research process as they are to be the focus of the observations [40,41]. If any patient declines to participate, their parents are excluded from the study.

#### 2. Data collection

A central assumption of ethnography is that to understand what people are doing and why: ...*one needs to understand the meanings involved: how they interpret and evaluate the situations they face, and their own identities *[35:168]. Communications between MDT members and parents is being explored using a combination of minimally obtrusive observations, verbatim field-notes and/or digital-recordings of planned and ad-hoc ward-based teaching/learning events, planned home-visits and outpatient appointments. Observations focus on key themes arising from a synthesis of data derived from phase 1. Individual, semi-structured, de-briefing interviews are also conducted with parents and professionals soon after selected observations, and selected case-note reviews are being undertaken. Interviews and reviews explore parents' and professionals' views and accounts of the effectiveness of observed communications. These data are supplemented with reviews of professionals' records of skills teaching and parents' skill and knowledge development, as well as parents' home-based clinical-management records. As data collection proceeds the inquiry becomes progressively focused on specific research questions. This allows for strategic data collection to pursue answers to the questions more effectively and to test these against evidence. Interviews are digitally recorded and transcribed verbatim.

#### 3. Data analysis

Data management is supported by Framework and Excel, analysis involves an iterative-inductive approach whereby the researchers move: *backwards and forwards iteratively between theory and analysis, data and interpretation*...[37:149]. This includes, for example, the analysis of identities and how these promote or hinder parents' learning. Based on prior literature [42,43] and our own recent research in this [17] we are interested in understanding more about the ambivalent identities parents and professionals adopt during care-giving, whether or not these are negotiated and how individuals engage with one another and relate to each other during care-giving practices and teaching/learning encounters. During preliminary analysis an initial coding frame will be devised comprising descriptive and analytic codes. This will be applied to the data set, and subsequently refined as analysis proceeds. The outcome will be emergent categories used to produce a description of the cases investigated, and could also include development of systematic typologies of professionals' parent-teaching interventions (plus the intervening researcher if appropriate) during shared care-giving practices.

### Ethical considerations

Approval was obtained from the North West 3 Research Ethics Committee (REC) (09/H1002/92), the University of Manchester REC and the participating NHS Trust R&D Departments. In both phases, obtaining informed consent is a key issue. After receiving written and verbal explanations and once any questions have been answered, participants who agree to participate provide signed consent and are assured of anonymity and confidentiality. We do not anticipate any risks or hazard to participating MDT members, patients or parents. It is possible that participants may feel they are being judged on their performance, to combat this, assurances are offered that the study is not 'testing' knowledge or 'judging' teaching, parenting or professional care giving skills but seeking an understanding of teaching interventions and communication styles that help parents to learn. It is also possible that observation periods could make participants feel uncomfortable; therefore, they are given a coloured card to use as a signal if they want the researcher to leave a situation, and are offered support via the Clinical Psychologist on the research team, if required. In line with ethical and legal guidance all identifiable personal information is handled in strict confidence. Parents' or professionals' individual views are not disclosed to each other or anyone else. Interview data are typed up as transcripts using a commercial transcription company. All person-identifiable information is removed from transcripts and field note data. No one other than the Chief Investigator and the researcher(s) appointed will be able to listen to the tapes or read notes or transcripts. The team may carry out further data analysis in the future, but the findings will always remain anonymised. All data will be stored in a secure, locked cabinet or password protected computer and will be available only to the researcher appointed and the Chief Investigator

## Discussion

Government policies and published research have consistently advocated home-based care for children with long-term conditions as a way of optimising their physical, emotional and social development [1,7,44-46]. To our knowledge this will be the first project to study the way MDTs teach parents to deliver home-based clinical care for their children in the children's kidney units in England, Scotland and Wales. The outcome of this study will be a detailed, in-depth analysis of professionals' parent-teaching strategies. This will provide a description of the cases investigated and may also include development of systematic typologies of parent-teaching interventions [37]. These outcomes will inform a phased approach to developing and evaluating an intervention that meets parents' learning needs. The study will provide a new understanding of MDT members' contributions to parents' learning in long-term kidney conditions [47]. The project is working with all children's kidney units in England, Scotland and Wales to make the results useful for future parent-teaching interventions and to inform the promotion of parents' competence. In addition, the outcomes will be made widely available to colleagues who educate health professionals. Our dissemination will include a funder's report, papers in peer reviewed journals and materials for distribution through parent networks.

### Potential limits of the proposed research

In keeping with mixed-methods' traditions and because of the small sample size and the condition-specific focus of the study, the findings are viewed as a 'snapshot' of the situation so will not be generalisable to other clinical settings. However, the results may serve as an important exemplar to inform parent-teaching in other long-term conditions so results may be transferable to other clinical contexts where parents undertake similarly complex, home-based clinical care of children with other childhood conditions (for example cancer or cystic fibrosis services). The focus of this study is on parents' learning although we do recognise that parents and older children may share knowledge about condition management; some children may also help their parents to understand complex aspects of treatments or may translate for them if parents' first language is not English. Nevertheless, parents are the primary care-givers and they have been selected as the primary focus for this study in order to generate knowledge about the context in which professionals teach parents.

### Future research

This study is part of a phased approach to developing and evaluating a complex intervention that meets the learning needs of families involved with UK children's kidney units in relation to home-based care-giving. Ultimately, a multi-disciplinary parent-teaching intervention to promote care-giving competence will be developed and subsequently this will be subjected to feasibility testing and piloting prior to evaluation through a randomised controlled trial, as indicated in guidance provided by the Medical Research Council on the development and evaluation of complex interventions. The current study will also produce concrete recommendations for developing future research involving children and young people learning to manage their own kidney conditions.

## Conclusions

This ongoing study uses mixed methods to survey multi-disciplinary teams' parent-educative functions in the 12 children's kidney units in England, Scotland and Wales. The findings will provide a unique and detailed insight into the context in which professionals teach and parents learn to manage home-based care of children's long-term kidney conditions. This will inform parent education, health professionals' educational curricula and health care policies.

## Abbreviations

MDT: Multi-disciplinary team; NHS: National Health Service; PI: Principle Investigator; REC: Research Ethics Committee; SPSS: Statistical Package for the Social Sciences

## Competing interests

The authors declare that they have no competing interests.

## Authors' contributions

VS conceived and initiated the study, VS and DA conceptualised and designed the study, JW, HL, TS, LW, LQ, JC and NJAW contributed to study design and conceptualisation and with VS and DA secured external funding; all authors approved the final version of the manuscript

## Pre-publication history

The pre-publication history for this paper can be accessed here:

http://www.biomedcentral.com/1472-6963/12/33/prepub
